# The Biological Value of Proteins for Pediatric Growth and Development: A Narrative Review

**DOI:** 10.3390/nu17132221

**Published:** 2025-07-04

**Authors:** Marlene Fabiola Escobedo-Monge, Joaquín Parodi-Román, María Antonieta Escobedo-Monge, José Manuel Marugán-Miguelsanz

**Affiliations:** 1 Department of Pediatrics, Faculty of Medicine, University of Valladolid, Avenida Ramón y Cajal, 7, 47003 Valladolid, Spain; jmmarugan@telefonica.net; 2 International Cooperation for Health and Social Development (CIDESS) Group, University of Las Palmas de Gran Canaria, Calle Juan de Quesada, 30, 35001 Las Palmas de Gran Canaria, Spain; 3 Valladolid Health Research Institute (IBioVALL), C. Rondilla Sta. Teresa, 47010 Valladolid, Spain; 4 Science Faculty, University of Cadiz, Paseo de Carlos III, 28, 11003 Cádiz, Spain; joaquin_parodi@yahoo.es; 5 Department of Chemistry, Science Faculty, University of Burgos, Plaza Misael Bañuelos SN, 09001 Burgos, Spain; antoitalia777@gmail.com; 6 Section of Gastroenterology and Pediatric Nutrition, University Clinical Hospital of Valladolid, Avenida Ramón y Cajal, 3, 47003 Valladolid, Spain

**Keywords:** animal-based proteins, plant-based proteins, micronutrients, amino acids, bioavailability, digestibility

## Abstract

In personalized nutrition, dietary guidelines must be adapted to the physiological and developmental needs of individuals across the lifespan, especially during childhood and adolescence. These should account for nutritional status, health conditions, and early-life risk factors, including those that emerge during pregnancy. This narrative review synthesizes recent evidence (2020–2025) on the biological value of protein sources in supporting pediatric growth and development. While adequate protein intake is essential for physical and cognitive development in individuals under nineteen, excessive intake may accelerate growth and increase the long-term risks of overweight and obesity. Compared to animal-based proteins (ABPs), plant-based proteins (PBPs) carry a higher risk of nutrient deficiencies in vulnerable populations due to lower digestibility and incomplete amino acid profiles. Although plant-based diets are encouraged for environmental reasons—particularly to reduce the ecological impact of livestock—protein intake must remain appropriate for age, sex, health status, and context. Nutritional strategies must ensure an adequate supply of essential amino acids and proper micronutrient supplementation, regardless of whether children follow diets rich in ABPs, PBPs, or a combination of both. Attention to these factors is vital to balancing nutritional adequacy with long-term health and sustainability goals.

## 1. Introduction

Nutrition has a critical role in human health, starting before conception and continuing throughout life. Pregnancy, childhood, and adolescence are crucial phases in human maturation, requiring well-balanced diets that promote individual well-being and environmental sustainability. In this framework, proteins are critical nutrients for growth, development, maintenance, reproduction, immune function, and overall health, especially in children and adolescents [1,2]. Dietary proteins provide nitrogen and AAs indispensable for synthesizing proteins, other nitrogen-containing compounds, and several metabolic routes [3]. They serve as precursors for active compounds such as neurotransmitters, glutathione, heme, and creatine. They play a vital role in the synthesis and function of muscles and organs, in producing enzymes and hormones, and in supporting the natural immunity of living organisms [4,5,6].

**Figure 1:**
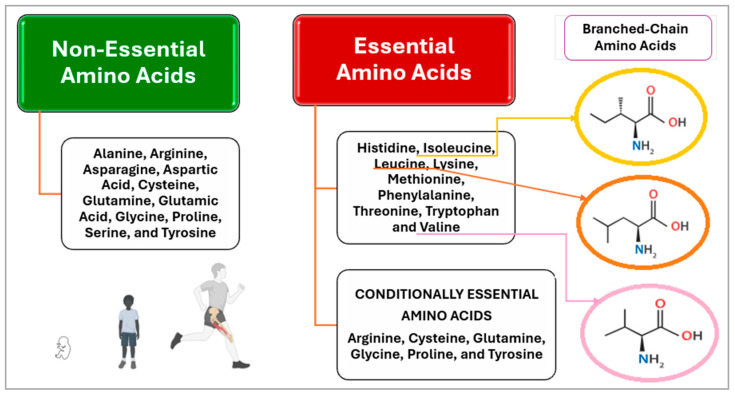
Types of amino acids according to their biological value.

Although the human body can synthesize dispensable AAs (DAA: alanine, arginine, asparagine, aspartic acid, cysteine, glutamine, glutamic acid, glycine, proline, serine, and tyrosine), also known as proteinogenic AAs, it cannot produce indispensable ones (IAA: histidine, isoleucine, leucine, lysine, methionine, phenylalanine, threonine, tryptophan and valine); these must be obtained exclusively through dietary protein intake. Nevertheless, various DAAs, such as arginine, cysteine, glutamine, glycine, proline, and tyrosine, may be converted into conditional IAAs for at-risk populations (infants, children, adolescents, pregnant and lactating females, and elderly); for example, premature infants need an adequate supply to counterbalance their insufficient synthesis (Figure 1) [7,8,9]. Considering this, plant-based proteins (PBPs) may offer a less complete AA profile than animal-based proteins (ABPs) due to lower digestibility and the AA deficiencies that vary by source. While these differences may be less significant for adults, they are crucial for the pediatric population due to their particular needs [10].

Personalized and precision medicine tailor treatments to an individual’s unique health status and living conditions; personalized nutrition (PN) surpasses the one-size-fits-all approach of traditional dietary guidelines by leveraging specific personal data. PN prescribes more precise and effective nutritional interventions—ranging from dietary advice to specialized products and services—designed to optimize health outcomes. Nutritional recommendations for PN should be tailored to individual needs at each life stage, taking into account their nutritional, health, and inflammatory status. Pediatric populations with chronic health diseases, such as cystic fibrosis (CF), have specific dietary needs to consider. In CF patients, we observed the risk of macronutrient and micronutrient deficiencies associated with their disturbed protein eating [11,12,13,14]. When evaluating protein intake in the pediatric population, critical factors are protein quality, quantity, AA profile, and whether the intake meets specific nitrogen and IAA requirements during specific physiological states in child development [15,16]. Therefore, this narrative review aims to compile the most recent findings on protein sources and their biological value related to the growth and development of children and adolescents.

## 2. Methods

In this narrative review on the biological value of protein sources, we conducted a literature search of articles published between 2020 and 2025 using electronic databases, including PubMed, Google Scholar, ScienceDirect, Web of Science, and the Cochrane Library. Inclusion criteria focused on individuals under 19 years of age, including pregnant adolescents. We excluded individuals over 19 years and pregnant and lactating women. To ensure a comprehensive search, we employed Boolean operators (“AND” and “OR”). Articles with English as the primary language, and all selected abstracts in English, regardless of the original language of publication, were included.

Initially, we used the keywords: “protein,” “biological value,” “vegetable,” “fruit,” “protein requirements,” “children,” “adolescents,” and “protein bioavailability.” This narrow combination of search terms and operators likely limited the scope of our results, yielding fewer than 30 relevant articles across the databases. Considering the significance of this topic, as acknowledged by organizations like the World Health Organization (WHO) and the Food and Agriculture Organization (FAO) of the United Nations, we broadened our search parameters.

The revised search strategy used the core terms: “protein,” “biological value,” “children,” and “adolescents,” along with complementary keywords including: “animal,” “plants,” “vegetables,” “legumes,” “fruits,” “other sources,” “alternative sources,” “needs,” “requirements,” “dietary,” “quality,” “amino acids,” “amino acid status,” “bioavailability,” “digestibility,” “linear growth,” “stunting,” “height,” “malnutrition,” “undernutrition,” “obesity,” “child development,” and “chronic diseases”.

## 3. Update on the Biological Value of Proteins

### 3.1. Understanding Biological Value in Proteins

Previous research states that a protein’s BV is crucial, as it measures how efficiently the body absorbs and utilizes its AAs for tissue synthesis. In addition, it encompasses several key nutritional factors, such as digestibility, the bioavailability of digested components, and the presence and proportion of essential AAs [17]. Based on animal models, dietary protein digestibility is assessed via the protein digestibility-corrected AA score (PDCAAS) and the digestible essential AA score (DIAAS) [7,18,19]. The PDCAAS calculation includes the WHO/FAO/UNU (United Nations University) Amino Acid Score (AAS), derived from the limiting AAs in meals. Specifically, this refers to the individual AA with the lowest concentration compared to the benchmark. If we multiply the FAO/WHO/UNU AAS by the protein digestibility from fecal nitrogen losses, we obtain the PDCAAS [19]. The DIAAS method also employs the FAO/WHO/UNU AAS, measuring nitrogen at the terminal ileum, which is key because colonic bacteria can affect fecal nitrogen levels and better reflect the actual amounts of AAs digested and absorbed [20].

Nonetheless, debate continues over the best strategy for obtaining information on dietary protein digestibility because, on the one hand, the PDCAAS has several issues. The PDCAAS method does not account for the additional nutritional benefits of proteins with a high BV. It tends to overstate the nutrient content and fails to recognize the presence of antinutrients in meals that can affect protein absorption. Furthermore, PDCAAS may incorrectly evaluate the digestibility of proteins from meals that naturally have low digestibility, particularly when supplemented with the corresponding limiting AA [20]. On the other hand, for human use of the DIAAS, experts recommend five research protocols related to DIAAS: evaluating the ileal absorption of AA in animal and human models; considering the real ileal digestibility of AAs; the application of a dual stable isotope tracer technique; the oxidation of a labeled indicator AA; measurements of postprandial protein use; and measurements of overall net postprandial protein application [16].

Additionally, the FAO of the UN Expert Working Group guided the assessment of protein quality in the Follow-up Formula for Young Children (FUF-YC) and Ready-to-Feed Therapeutic Foods (RUTF). They recommend using PDCAAS with existing protein digestibility values or the DIAAS when individual AA digestibility values are available to assess protein quality. To estimate digestibility coefficients (of protein or AAs), a flow diagram of actual ileal AA digestibility values in humans, growing pigs, and rats was proposed. If this is infeasible, they recommend using fecal protein digestibility values. A PDCAAS index of 90% or higher is considered adequate for these formulations. If the index is below 90%, we must increase the protein content to meet the requirements [21]. For DIAAS value measurement, the FAO suggests using specific scoring patterns [7]. Table 1 compares different protein sources based on their biological value, digestibility, net protein utilization, and amino acid content.

Another crucial aspect regarding AA digestibility is the FAO Expert Consultation on the Quality Assessment of Dietary Proteins in Human Nutrition reports, which consider each AA as an individual nutrient [20]. It is essential to recognize that human nutritional requirements are not for protein but for nitrogen and the nine IAA. Protein is the primary source of nitrogen and AAs in daily nutrition [9]. Human protein intake requirements refer to a protein source with a complete IAA profile that is fully digestible (DIAAS ≥ 100). However, numerous proteins do not meet these requirements due to a deficiency in one or more IAA, or because they are not easily digested [7]. Except for lysine and threonine, we can meet the metabolic needs of IAAs because the body can produce other IAA via transamination reactions by ingesting their keto acids [8]. Proteins with a BV close to 100, such as egg protein (BV ~100) and whey protein (BV ~104–110), are highly efficient sources (Table 1). Most PBPs have lower BVs when compared to ABPs because they often lack one or more essential AAs [32].

We need to be cautious when using PBP sources because of their potential antinutritional factors (ANFs) and bioactive compounds, particularly inhibitors such as trypsin, tannins, phytic acid (or phytates), hemagglutinins, glucosinolates, and gossypol. These substances disrupt protein digestion by interfering with digestive enzymes, blocking nutrient absorption of the intestinal epithelium, binding to meal proteins and causing them to clump, or binding to and inactivating nutrients, digestive enzymes, and mineral cofactors [21,22]. The processing methods applied to foods influence their digestibility and alter amino acids, which impacts protein quality [7]. Although we can improve the digestibility and bioavailability of PBPs by numerous appropriate processing technologies, such as physical, chemical, and biological approaches containing sonication, microwave, high-pressure processing (HPP), and field electric (EF), as well as enzymatic hydrolysis and fermentation [33], the quality of the absorbable protein or AAs will depend on the source (vegetable and fruit) [34]. We should adopt proper treatment measures to minimize the effects of food processing and storage that can generate ANF, e.g., the Maillard reaction, racemization, and lysinoalanine. Moreover, intense heat treatment (130 °C for 45 min) reduces the standard ileal digestibility of all AA. Methionine and cysteine are vulnerable to oxidation throughout the process and through the protein crosslinking between the ε-amino group and the acidic AAs [21].

### 3.2. Protein Requirements for Human Growth and Development

**Figure 2:**
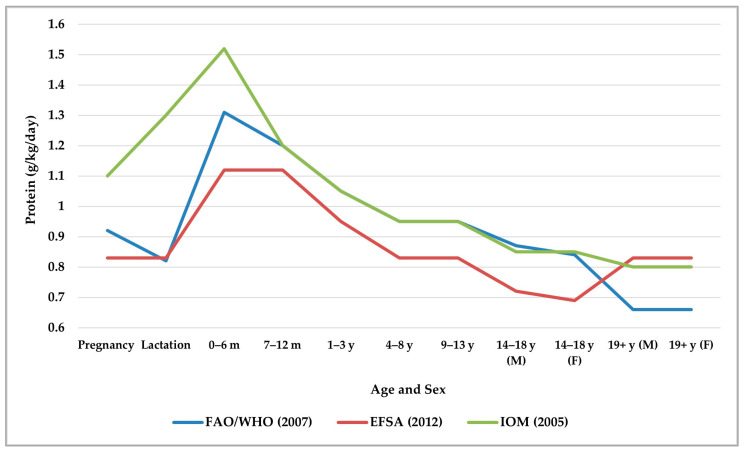
Comparison of the recommended dietary intake of protein between the Food and Agriculture Organization of the United Nations/World Health Organization (FAO/WHO), the European Food Safety Authority (EFSA) and the International Medicines Organization (IOM) by age group and sex [28,35,36].

In pediatric growth and development, it is key to note that the total daily protein recommendation is an estimation derived from average body weight and moderate levels of physical activity. For infants (0–6 months), the FAO of the UN Expert Working Group used the AA composition of breast milk. For children aged 0.5 to 3 years, the scoring patterns result from the values applicable to the 0.5- to 1-year age group. For children under 3 years, teenagers, and adults, they use the scoring patterns group of 3–10 years of age [7]. In addition, the FAO of the UN Expert Working Group recommended, for the age category of 1 to 2.9 years in FUF-YC, a protein requirement of 0.86 g/kg/day, along with corresponding AA requirements; in the case of RUTF, a protein requirement of 2.82 g/kg/day was recommended, to support a catch-up weight gain of 10 g/kg/day in children with severe acute malnutrition [21]. Figure 2 summarizes and compares the RDI of protein by age group and sex, according to recommendations from international organizations such as the FAO/WHO/UNU Expert Consultation [28], the European Food Safety Authority (EFSA) [35], and the International Organization of Medicine (IOM) [36].

The amounts shown in Figure 2 are the minimum recommended intakes to maintain nitrogen balance in healthy subjects. International organizations have varying recommendations for protein intake during pregnancy, lactation, and the first six months of life. Increasing protein intake during pregnancy and lactation is essential to provide the necessary protein for fetal development and milk production. The WHO recommends exclusive breastfeeding for the first six months of life to promote optimal growth and development. We should ensure that non-breastfed infants receive a protein intake of 1.5 to 2 g/kg/day. Between 1 and 18 years of age, we observe a gradual reduction in protein recommendations, which are quite similar between FAO/WHO and IOM but lower in the EFSA guidance. In adults, while FAO/WHO establishes a minimum recommendation of 0.66 g/kg and a safe level of 0.83 g/kg, the IOM and EFSA recommend a similar protein intake of around 0.8 g/kg. In Europe and the USA, there is consensus in recommending 0.8–0.83 g/kg of protein as an adequate intake for adults. In older adults and people with chronic diseases, the protein requirement may increase to 1.0–1.2 g/kg/day to preserve muscle mass [28,35,36,37,38].

**Figure 3:**
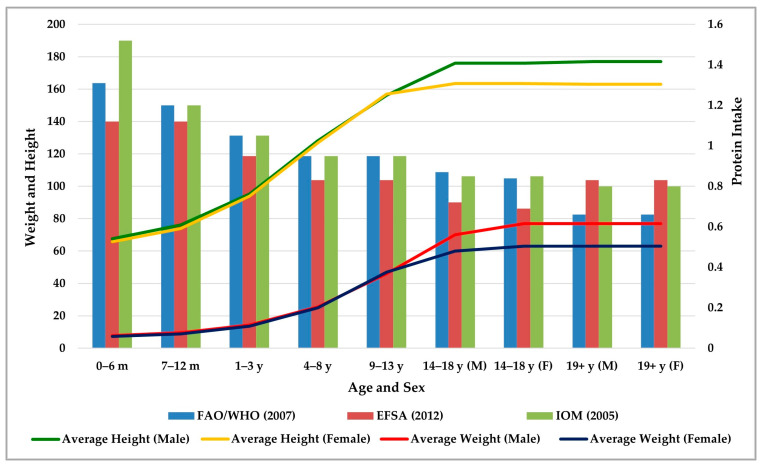
Relationship between recommended dietary intake of protein in g/kg/day of the Food and Agriculture Organization of the United Nations/World Health Organization (FAO/WHO), the European Food Safety Authority (EFSA), and the International Medicines Organization (IOM) with average height (cm) and weight (kg) by age group (months for infants under one year, years thereafter) and sex [male (M) and female (F)] [20,35,36,40,41].

In human nutrition, from conception to age 2, children experience a critical period of growth and development [39]. Growth accelerates from birth to age three, especially in the first year of life. Growth then remains constant until age eight (5–6 cm per year). The pubertal growth spurt typically begins between the ages of nine and thirteen, occurring earlier in girls, who tend to stabilize their height sooner than boys. Boys experience substantial growth between the ages of 14 and 18. Finally, adult height is typically reached, with minimal variations, between the ages of 19 and 25 [40,41]. Protein intake is related to the nutritional requirements for the growth and development of children and adolescents. Figure 3 presents a comparison between the RDI of protein suggested by the aforementioned international organizations and average heights and weights by age and sex group. Even though protein intake recommendations decrease with age, height and weight increase. However, protein consumption is higher because body weight augments with age. It is vital to emphasize the strong connection between age- and sex-specific protein and AA requirements and growth trajectory, as growth velocity and weight gain can be compromised by protein-calorie malnutrition, resulting not only in growth retardation and even stunting and wasting but also in immune dysfunction, with a subsequent risk of acute and chronic diseases [39,42].

It is crucial to know the link between the dietary habits of pregnant women and adverse outcomes in newborns, including gestational diabetes mellitus, preeclampsia, restricted fetal growth, low birth weight, small for gestational age infant, etc., [43,44]. These conditions, together with an inadequate period of breastfeeding in the first six months and complementary feeding in the first 2 years, can affect not only weight gain, height, and neurological development but also augment the subsequent risk of developing chronic illnesses throughout life such as obesity, diabetes mellitus (DM), metabolic syndrome (MetS), cardiovascular diseases (CVDs), etc. [45,46]. In addition, many nutrition-related chronic diseases (NRCD) are increasing worldwide, especially in developed countries, and may appear in childhood. Currently, in children, we must face the triple burden of malnutrition (TBM), which includes not only undernutrition—stunting and wasting—and overnutrition—overweight and obesity—but also micronutrient deficiency or the so-called hidden hunger [45,46]. We found a higher risk of deficiencies in macronutrients and micronutrients among pediatric patients with chronic diseases related to their protein intake [47,48,49,50,51,52]. Another foremost aspect of understanding the mechanisms of the double burden of malnutrition (DBM) in childhood requires recognizing the transition patterns typical of each life stage. This awareness is crucial for creating targeted interventions that effectively support recovery [53]. Protein intake differs significantly worldwide, especially in low- and middle-income countries (LMICs), being substantially lower than in high-income countries (HICs), particularly for protein from ASF [2,16].

Children who consume foods with insufficient amounts of IAA have a higher incidence of stunting. Examples include typical diets that lack animal-source foods that may fall short of meeting daily IAA requirements. An East African diet composed of 80% cassava and 20% common beans does not provide proper IAA intake. In contrast, a West African diet consisting of 80% maize and 20% cowpeas, as well as a South Asian diet with 80% rice and 20% lentils, can meet the IAA requirements for both healthy children and those with environmental enteric dysfunction (EED) but are insufficient to support catch-up growth [39]. EED is a subclinical disorder characterized by malabsorption and increased small intestinal permeability, which is caused by villus atrophy, crypt hyperplasia, and immune cell infiltration, leading to growth impairment [54]. Therefore, it is vital to know the critical moments of child maturation to avoid and reduce the occurrence of DBM and TBM. A Young Lives cohort study conducted in three LMICs (India, Peru, and Vietnam) examined the transition from normal to stunted, overweight, and concurrent stunting and overweight (CSO). The authors observed that early childhood prevents CSO and overweight individuals, while puberty is crucial for reversing them. CSO often appears in late teenage years and early adultness and progresses primarily through growth retardation or, secondarily, overweight, with little setback potential [53]. These results may indicate key moments to act.

A healthy diet promotes growth and development and prevents malnutrition [7]. Global estimates indicate that 22.3% of children under 5 years old (148.5 million) suffer from stunting, 6.8% (45 million) undergo wasting, and 5.6% (37 million) are carrying extra weight [55]. Pediatric populations in LMICs are at a significant risk of consuming inadequate levels of IAAs and usable dietary protein for growth [39]. In LMICs, stunting is frequent and associated with increased child morbidity and mortality, poor cognitive and motor development, poor academic and intellectual performance, lower economic productivity, and decreased future income. While children generally consume enough total protein, their intake of IAA often falls short due to a lack of dietary diversity and low protein quality. During this crucial developmental phase, it is essential to ensure adequate IAA intake by incorporating nutrient-dense animal-source foods to help address potential growth issues that may arise during complementary feeding [39]. In addition, it is decisive to note that babies and toddlers typically progress from a stable state of health to an acute infectious state during routine infections, followed by a rapid recovery phase after experiencing an illness or food insecurity [21,56]. Growth recovery requirements may overlap with additional necessities during the recovery period from infection. Nitrogen losses will depend on the intensity and duration of the disease. Experts recommend a 20–30% increase in total protein (30–50% in the case of diarrhea) for a recovery period twice to three times longer than the duration of the disease [57,58].

In the pediatric population, many diseases require a differentiated protein intake, for example, in children with chronic kidney disease (CKD), in addition to maintaining normal body mass and composition, minimizing comorbidities, slowing disease progression, and reducing long-term mortality and morbidity; a specific additional goal for protein intake is to keep standard maturity. Their poor progress is associated with increased hospitalizations and higher mortality in end-stage CKD (ESKD). Therefore, to prevent excessive calorie intake, we need to adjust energy intake based on lean body mass and align protein intake with ideal body weight, ensuring that malnourished children receive adequate nutrition. To promote optimal growth in children with CKD aged 2 to 5 years, the goal is to achieve the upper limit of the RDI of protein. For children on dialysis, protein intake should be even higher to compensate for losses that occur during the dialysis process. For consistently high blood urea nitrogen levels, we should reduce protein consumption to the minimum of the suggested dietary intake (SDI) to prevent metabolic acidosis correlated with the advance of chronic kidney disease [59]. Additionally, during infancy, some children develop cow’s milk protein allergy (CMPA), the most prevalent food allergy in this age group, affecting between 1.9% and 4.9% of infants under one year. These children often require hypoallergenic formula substitutes for breast milk such as extensively hydrolyzed formulas (CMP-eHF) or amino acid-based formulas (AAF) [18]. Other nutrition-related diseases include celiac disease, caused by an allergy to gluten, a protein found in wheat [60].

In childhood cancer, malnutrition increases the risk of negative consequences, lower tolerance to cancer therapy, elevated mortality, and an augmented risk of relapse in comparison with those with an adequate nutritional status and a diet balanced in macro- and micronutrients. During childhood growth and development, protein is essential for new tissue synthesis, such as muscle tissue, and contributes to the repair of tissue damage due to cancer treatment. For example, in youths undergoing hematopoietic stem cell transplantation (HSCT), increased metabolic demands and reduced oral consumption make it challenging to maintain adequate protein levels, leading to an imbalance between protein synthesis and degradation. Protein hypercatabolism can be cumulative if there was prior malnutrition, producing a loss of lean body mass that affects growth and development. Graft-versus-host disease (GVHD) is associated with protein-losing enteropathy via exudative activity. Even though there is no consensus on protein recommendations for these patients via enteral and parenteral nutrition, in an international reference survey and exploratory review on the protein requirement of patients with HSCT, the authors found that some of them suggested an amount of protein (by age group) marginally above the protein intake range advised by ASPEN for critically ill pediatric patients, which spans from 1.8 to 3.0 g/kg/day. In other cases, the authors defined amounts between 1.0 and 3.0 g/kg/d by phases (maintenance and stress) according to age [61].

### 3.3. Protein Sources

Regarding protein sources, it is essential to acknowledge that animal and marine-derived proteins, including meat, dairy, eggs, and fish, supply all critical macro- and micronutrients required for proper growth and development. Meat is a rich source of protein with a high BV, containing all nine essential amino acids and vitamin (Vit) B12. Dairy and poultry products are excellent due to their high protein digestibility and favorable amino acid profile. Habitual intake of eggs increases skeletal muscle mass. Consumption of these proteins is safe and can meet most nutritional requirements when ingested in suggested amounts as part of a typical diet [62]. More than 80% of the world’s population consumes meat due to cultural factors, gender, geographic location, availability, and affordability [63]; however, globally, many people have a restrictive diet related to ABPs for cultural, religious, or socioeconomic reasons. These meals include flexitarian, vegetarian (lacto–ovo and lacto), pescatarian, vegan, and macrobiotic diets [64]. Nutritional critics argue that PBPs are nutritionally incomplete because they are deficient in Aas such as lysine, valine, branched-chain AAs, and the sulfur-containing AAs, methionine and cysteine (except for grain proteins) [65].

It is essential to understand that animal-based foods (ABFs) are crucial for providing vital nutrients necessary for growth and development during childhood, including iron, calcium, zinc, selenium, riboflavin, Vit-A, and B12. Fish and seafood are particularly rich in Vit-D, iodine, and long-chain omega-3 fatty acids such as EPA and DHA. On the other hand, plant-based foods (PBFs) offer valuable polyunsaturated fatty acids (PUFAs), especially the essential fatty acids α-linolenic acid (ALA) and linoleic acid (LA). A systematic review of 50 studies conducted between 2000 and 2022 examined children aged 2 to 18 years. The review found that while the average protein intake met recommended levels across all dietary patterns, those following plant-based diets had a lower protein intake. Vegetarians had an average protein intake of 12.5%, while vegans had a lower average of 10.9%. In contrast, meat-eating children had a protein intake of 13.8% [66]. Although omnivores may have a suboptimal level of vitamin D and iodine in childhood and puberty [67,68], deficiencies in protein, Vit-A, B2, B12, selenium, and calcium are considered crucial in vegan diets. In vegan diets, and in vegetarian diets, deficiencies of iron, zinc and long-chain n-3 fatty acids [64,68,69] are observed. Furthermore, vegan children are particularly susceptible to vitamin D and iron deficiency due to an inadequate supply and/or dietary fiber excess and other components that limit their bioavailability [64,69].

We are facing a global challenge in providing adequate essential nutrition to pediatric populations [70]. However, a study called the VeChi Youth study, which involved 401 children aged between 5.5 and 19.1 years, found no significant dietary risks associated with vegetarian and vegan children compared to their omnivorous counterparts [68]. While ABF are safe and important in an omnivorous diet, PBF lack vitamin B12 and have lower biological value and anabolic effects. Therefore, vegetarians should consume fortified foods or B12 supplements to ensure adequate nutrition [70]. Although a plant-based diet can provide nutritional benefits, restrictive plant-based diets can lead to nutrient deficiencies and increase the risk for vulnerable population groups, especially in infants, children, adolescents, pregnant and lactating women, and older people, by decreasing nutritional flexibility and robustness [70,71]. In vegetarian and vegan diets, the more restrictive the diet and the younger the child, the greater the risk of suffering malnutrition related to the quantity and quality of proteins and other nutrients [64,69]. Furthermore, a poor supply of high-quality protein can cause negative consequences, particularly for those with augmented nutrient needs such as children in nutritional recovery [71]. Vegetarians and vegans should enhance their consumption of soy, nuts, legumes, and seeds to ensure the proper intake of protein, iron, and zinc [72].

The Dietary Guidelines for Americans (DGA) 2020–2025 use the ounce-equivalent (oz-eq) as a standard unit to represent foods from the protein group that offer comparable nutritional value. For instance, one oz-eq corresponds to one ounce of meat, one whole egg, 0.25 cups of beans, or 0.5 ounces of nuts [73]. However, Connolly et al. report that animal- and plant-based protein sources do not deliver the same levels of indispensable amino acids (IAAs) or postprandial bioavailability needed to support protein synthesis in both young and older adults [74] From twenty-three studies that used dietary models to forecast the protein influence of PBPs, the authors observed that although protein eating from PBP simulations was lower than that from animal-based diets, it met the specific nutrient requirements of each country. However, some imitation diets for children and older adults did not meet protein adequacy for specific PBFs. In certain studies, researchers observed a decline in AA adequacy as the intake of PBFs increased—particularly depending on the types of foods used as substitutes. Legumes, nuts, and seeds offered higher protein quantity and quality than cereals. However, completely replacing ABFs with PBFs led to lower protein adequacy compared to both baseline diets and partial substitutions [71].

### 3.4. Growth and Development with Regard to Protein Consumption

Numerous studies have tried to explain the impact of protein intake on child growth and development. Consuming an adequate amount and quality of proteins in the diet is critical for growth. However, a diet that is excessively elevated in protein can lead to increased speed growth and augment the risk of being overweight and obese both during childhood and later in life. In a study involving 345 children followed at 9 months and again at 5 years, intake of non-dairy animal protein (NDAP) during infancy—unlike dairy or plant-based protein (PBP)—was linked to a higher body mass index (BMI) z-score in early childhood [75]. Furthermore, there was an association between increased total and ABP consumption during infancy with extreme BMI in youth and adolescence [75,76,77,78]. Even though there is evidence suggesting a correlation between elevated protein consumption in childhood and an augmented risk of obesity later in life, an elevated protein intake may contribute to a higher fat-free mass index (FFMI), which can promote muscle development and total body composition, and greater satiety, which can help to control appetite and reduce calorie intake, thus contributing to the maintenance of a healthy weight. The overactivation of growth pathways and elevated insulin-like growth factor-1 (IGF-1) levels (2) may explain this effect [2]. It is essential to note that the adequate intake of AAs may reduce risk areas and maintain a child’s health.

It is imperative to note that the Longitudinal Studies of Child Health and Development at the Boston Lying-in Hospital followed 131 individuals (67 females) from birth to adulthood. Researchers observed a correlation between higher fat and ABP eating, especially at ages 9 and 10, and with earlier and higher peak growth velocity (PHV) and greater height at age 13 and in adulthood. In contrast, they observed a correlation between higher PBP eating between ages 9 and 10 and later PHV and higher PBP from ages 1 and 10 with a shorter height at age 13 [79]. Moreover, in a systematic review (14 articles) and a meta-analysis (eight articles), animal-source food supplementation in children (aged 6 months to 2 years) resulted in a substantial effect size on length-for-age (LAZ, 0.15) and weight-for-age (WAZ, 0.20) z scores, in comparison to the control group. The authors found the highest effect sizes for LAZ (0.31) and WAZ (0.36) with egg intake. Eggs may have produced this effect due to their well-balanced composition and high-quality protein, distinguishing them from other ABFs. Furthermore, their leucine and glutamine content appear to regulate the mTORC1 activation pathway in skeletal muscle protein synthesis [80].

Additionally, in 187 prepuberal Polish children (34 omnivores, 63 vegetarians, and 52 vegans), the results showed that, in contrast to omnivores, vegan children had reduced cholesterol and high-sensitivity C-reactive protein (hs-CRP) levels; lower cardiometabolic risk; lower fat mass; shorter height; lower bone mineral content (BMC); and micronutrient deficiencies. Vegetarians exhibited a less optimal profile of cardiometabolic risk factors. Limiting animal products may affect children’s growth and bone health, potentially leading to deficiencies in Vit-B12 and D. Thus, these individuals require proper supplementation. In addition, since atherosclerosis begins in childhood and becomes a risk factor for CVD in adulthood, vegan diets with a better CVD profile could potentially contribute to reducing its prevalence [81]. In contrast, longitudinal studies on bone growth have shown that adequate amounts of PBPs from a single source and formulation can perform comparably to casein, the recognized gold standard [63]. Nevertheless, a cross-sectional study involving 3299 Chinese children and adolescents (aged 6–18 years) reported a negative association between protein intake—both in terms of dose–response and as a proportion of total energy intake—and linear growth. There were no differences in the protein sources [1].

Another significant aspect of protein intake is its relationship with gut microbiota and growth. For instance, a prospective cohort study in children (6–8 years) included 1826 adolescent participants to analyze the gut microbiome associated with dietary protein in a cross-sectional study. The authors classified participants into tertiles according to protein intake. Children in the highest animal protein-microbial index (APMI, *unidentified_Saccharomonas*) tertile (the mean intake of 59.4 g/day) were more likely to experience earlier menarche or voice break than those in the lowest APMI tertile. In contrast, children with an elevated plant protein-microbial index (VPMI, *Butyricicoccus, Enterococcus*, *Dorea*, and *Romboutsia*) were presumed to reach voice break or menarche later than those with a minor VPMI. While VPMI explained 39% of pubertal onset, APMI explained 15% of early pubertal onset. This difference could be due to its digestibility. PBA is easy to digest, resulting in minus degradation by intestinal bacteria, while PBV, with minimal digestibility, depends more on intestinal metabolism [82].

Conversely, although a low protein intake can limit growth, high protein consumption during early childhood can lead to rapid weight gain and an augmented risk of overweight and obesity due to increased body fat mass. This connection is more distinct in ABP compared to PBP. In addition, some researchers found (in adults) an association between plasma protein metabolites (protein intake intermediate phenotype) and DNA methylation [83]. These associations could be due to the effects of AAs, especially branched-chain AAs (BCAAs), and other AAs, such as arginine, which stimulate insulin and IGF-I secretion and influence preadipocyte metabolism, leading to overweight in children [83,84]. In a meta-analysis of European ethnicity cohorts, an association was observed between childhood PBA intake and DNA methylation (cg21300373 and cg10633363) in late youth (between 7 and 12 years), and an association between childhood PBP intake and DNA methylation (cg25973293 and cg15407373) in early youth (between 2 and 6 years). Variations in protein quality and the supply of methyl donors could modulate site-specific CpG methylation patterns [83,85], a universal methyl donor S-adenosylmethionine precursor, which might change the DNA methylation pattern, especially at specific loci [83,86].

### 3.5. Alternative Proteins

Interestingly, another critical factor in individual and population nutrition related to protein intake is the bioeconomy, which uses renewable biological resources from land and sea, such as crops, forests, fish, animals, and microorganisms, to produce food, materials, and energy [87]. Interest in the bioeconomy has surged in recent years, driven by shifting consumer preferences, the urgent need for sustainable strategies to nourish a projected global population of 11 billion by 2050 [16,88,89,90], and growing environmental and ethical concerns related to conventional protein production methods [86,87,88]. In terms of greenhouse gas emissions (GGE) and land use, diets high in meat have an environmental footprint that is four times greater than that of a vegan diet, are nearly three times higher in terms of biodiversity loss, and twice as high in water usage [91,92]. Although expanding livestock production may help to boost the global protein supply, it could come at a severe environmental cost, including higher GGE, pollution of water and soil, and intensified biodiversity loss [90]. Experts emphasize that transitioning toward plant-based diets is a promising approach with which to mitigate the environmental impacts of animal agriculture [91,92]. Nonetheless, it remains essential to ensure that both individual and population-level dietary protein needs support global food and nutrition security [93]. Adjusting protein quality at the population level significantly reduces the environmental footprint difference between foods of animal and plant origin [71].

The most significant criticism occurs when assessing the nutritional value of a protein source. It is crucial to compare the protein adequacy of animal and plant sources by considering AA profiles, digestibility, and bioavailability as key indicators of protein quality [91,92]. This key also applies to alternative protein sources, a subset of the bioeconomy that includes microbial proteins (e.g., microalgae, mycoproteins), ABPs, cultured meat, plant-based alternatives to meat and dairy, and fungal proteins. Some proteins are traditional but undervalued, such as the *Emerita analog* (Muymuy in Peru) [94], while others are biotechnological innovations [88,89]. It is essential to recognize that some conventional and undervalued foods, like tofu and various edible insect species, are being introduced to new geographic areas. In addition, emerging strategies include plant-based meat substitutes and biotechnological advancements such as cell-cultured production [95]. Nonetheless, we must ensure the efficacy and safety of these alternative proteins for widespread consumption within an appropriate regulatory framework. Similar to traditional protein sources, the nutritional value of these emerging protein alternatives and high-protein products will differ based on their overall protein composition, AA profile, and digestibility [16].

The PBP sector—particularly products designed to replace conventional meat and dairy—has grown in response to global concerns about the health and ecosystem impacts of animal product consumption. The rising demand for non-animal protein sources has given rise to so-called “flexitarian” consumers—individuals who intentionally reduce their intake of meat, dairy, and eggs in favor of PBFs, motivated by environmental concerns, health benefits, or both [96]. In addition, nutrition experts are advocating for flexitarian diets, which encourage greater incorporation of PBPs and could lead to a 25% reduction in global animal product consumption [90]. While PBP requires less land (38–91%), water (53–95%), and carbon emissions (69–92%) than animal proteins, incorporating alternative proteins into European diets has the potential to cut global warming impacts, as well as water and land usage, by over 80%. These protein sources are attractive alternatives that can partially replace protein obtained through ecologically harmful practices like factory farming or the overexploitation of fisheries [88,89].

Additionally, the growing popularity of people consuming meat-free diets in industrialized countries, such as vegetarian diets that exclude meat and fish and vegan diets that eliminate animal products, dairy, and eggs, arises out of concerns for planetary sustainability, improving health, reducing the risk of non-communicable diseases (NCDs), and considerable concern for animal welfare [81,97]. In terms of overall food expenses, the vegetarian diet proved to be the most cost-efficient, regardless of total daily energy intake (TEI). Across all dietary patterns, the substantial contributors to daily food costs were starchy foods, fruits, snacks and sweets, beverages, and vegetables. Among omnivores, meat, and fish products also represent a significant portion of the cost. Protein-rich foods—including legumes, nuts, seeds, dairy and meat alternatives, dairy products (for vegetarians and omnivores), and meat/fish (for omnivores only)—accounted for approximately 25% of total food spending across all groups. In the VeChi Youth Study, the value of meat-based foods was comparable to the sum of the value of PBP sources, such as legumes, nuts, seeds, and meat alternatives, in vegan diets [98].

## 4. Discussion

This narrative review aims to present the latest research on protein sources and their biological value in the growth and development of children and adolescents. Our research is novel and unique, as it encompasses not only an understanding of protein quality measurement, digestibility, bioavailability, and requirements during human growth and development, but also traditional and alternative protein sources and their impact on society and the environment. Furthermore, this review highlights future perspectives on amino acids and the essential role of proteins in the context of personalized nutrition, emphasizing the contribution of omics technologies, bioinformatics, and their impact at the cellular level. It is crucial to recognize that PBFs can be high in protein, more affordable, and environmentally sustainable compared to ABF, which provide highly digestible and bioavailable amino acids. Nevertheless, FANs and certain types of fiber can affect the digestion and absorption of AAs, which, if not properly corrected, can lead to an underestimation of protein quality. In addition, although some processing methods may improve protein solubility or shelf life, they often result in a reduction in the bioaccessibility or bioactivity of AAs, ultimately decreasing their nutritional value.

From a dietary perspective, relying solely on traditional protein quality metrics, such as the PDCAAS, is increasingly insufficient, as it fails to fully capture the complexity of human nutritional patterns, particularly the diverse combinations of proteins consumed in meals. The DIAAS offers a more accurate assessment by taking into account ileal IAA digestibility [99]. When using the PDCAAS, it is essential to rectify the digestibility of individual IAAs. This correction should account for specific endogenous IAA losses related to the fiber class and NAF present in the protein source. Alternatively, actual ileal IAA digestibility values obtained using techniques that consider the inhibitory effects of NAFs in the food matrix can be used [8,21]. Using the PDCAAS without adjusting for endogenous IAA losses associated with specific food matrix components further limits its usefulness. Conversely, methods, such as the direct measurement of ileal digestibility, the dual stable isotope tracer approach, or the Indicator Amino Acid Oxidation (IAAO) technique, provide more robust and physiologically relevant data by incorporating the inhibitory effects of ANFs [18,21]. Inadequate IAA content can limit protein synthesis [22], especially in vulnerable populations such as children and adolescents. Therefore, this highlights the urgent need for a refined protein quality assessment system that reflects not only the origin (animal or plant) but also the synergistic effects of mixed dietary protein sources [19]. Without such a nuanced approach, nutritional recommendations risk being oversimplified or nutritionally insufficient.

Although current guidelines recommend high protein intake for older adults to preserve muscle mass and prevent frailty, the effects of elevated protein consumption during childhood and adolescence remain controversial. Children are generally advised to consume between 0.95 and 1.3 g of protein per kilogram of body weight per day, depending on age and individual needs. However, in many developed countries, children and adolescents often consume protein at levels two to three times higher than the RDA. This excess raises concerns about potential long-term health implications, which are not yet fully understood and may involve both benefits and risks [2]. A meta-analysis of 62 trials (>30,000 participants) across five continents found that animal protein supplementation during pregnancy and early childhood increased child weight and reduced the risk of stunting. Supplementation in infancy (but not formula) modestly improved height-for-age. Researchers did not observe growth benefits in preterm infants. Given the limited high-quality data, conclusions about linear growth remain uncertain. Nonetheless, balanced protein-energy supplementation remains beneficial for recovery from wasting and may offer additional advantages, such as cognitive development, warranting further investigation [100].

In healthy, weight-stable adults, the mean protein requirement is 0.66 g/kg based on nitrogen balance studies, with a Population Reference Intake (PRI) set at 0.83 g/kg/day [15]. Nevertheless, protein requirements in children and adolescents are more complex and are shaped by factors such as growth rate, sex, and physical activity. Experts calculated these needs using various methods, including nitrogen balance, and, more recently, stable isotope techniques. Current recommendations suggest intakes of 1.2, 1.05, 0.95, and 0.85 g/kg/day for children aged 7–12 months, 1–3 years, 4–13 years, and 14–18 years, respectively [101]. Recent studies challenge these values. For instance, a 2021 study by Hudson et al., with a more precise Indicator IAAO method, proposed that children aged 6–10 years may require up to 1.55 g/kg/day—significantly higher than current guidelines. This increase in protein needs could be related to physical activity [102]. This discrepancy highlights the limitations of older estimation methods and suggests that the current RDI might underestimate correct physiological needs, particularly for active children.

PBPs have the potential to serve as viable alternatives to ABPs, provided that new PBP complexes with improved properties use advanced processing strategies. Emerging approaches, such as combining protein–protein interaction technologies to enhance functional properties with fermentation or germination techniques to improve nutritional quality, show promise. However, current PBP-based foods still have significant drawbacks, such as the presence of allergens and off-flavored compounds, which compromise both sensory appeal and safety, limiting their broader application in the food industry [103]. Despite their potential, most research and innovation in the PBP sector focuses strictly on protein fortification and supplementation, often ignoring the structural and metabolic limitations of these proteins. For example, although PBPs can contribute to the development of lean body mass during exercise training, especially when intake exceeds 30 g per meal, their anabolic potential remains lower than that of ABPs [22].

Additionally, Alam et al. (2025) asserted that while adequate protein intake provides clear physiological benefits—supporting muscle development, immune function, and hormone production—excessive intake may impair kidney function, particularly in individuals with pre-existing renal conditions [2]. In high-resource settings and among affluent populations, concerns have emerged over the overconsumption of ASF (meat, dairy, and eggs), which are related to a higher risk of NCD even in children, including obesity, CVD, hypertension, type 2 diabetes, renal impairment, and prostate cancer. However, invoking these concerns as a justification for limiting ASF intake in LMICs, marginalized communities, or nutritionally vulnerable populations—especially during the complementary feeding period—is both scientifically flawed and ethically problematic. Blanket restrictions or recommendations to reduce ASF intake in these contexts risk exacerbating existing nutritional deficits and health disparities. In resource-poor settings, where micronutrient deficiencies and PEM are widespread, ASF plays a critical role in meeting essential nutrient requirements—such as high-quality protein, zinc, and bioavailable iron—particularly for infants and young children, who may lack access to exclusive breastfeeding [80]. For these populations, ASFs are not optional; they are indispensable.

On the other hand, although several studies have explored protein intake in children and adolescents, current evidence is insufficient to justify a broad revision of AA requirements or up-to-date reference standards. Still, this data gap must not lead to inaction. There is an urgent need for high-quality research focused on vulnerable groups—particularly infants and older adults—whose nutritional needs remain poorly characterized. This research gap disproportionately affects LMIC and small nations, which lack sufficient resources to conduct independent studies on protein quality. In this context, the role of organizations, like the FAO, is essential; providing open-access databases on the digestibility and nutritional quality of diverse protein sources—including novel and climate-resilient options—helps to democratize knowledge and support evidence-based decision-making in food and agriculture [16,104]. However, data alone are insufficient without the institutional capacity to apply the data effectively in public policy.

According to recent FAO estimates, the increased production and consumption of alternative proteins could significantly contribute to ecosystem restoration and reduce the carbon footprint of agri-food systems, which are responsible for 31% of anthropogenic GGE. However, this transition to a new protein matrix should not be driven solely by environmental arguments without a rigorous assessment of their nutritional value, impact on human health, and implications for food security. The indiscriminate promotion of alternative proteins risks reproducing the errors of the dominant food model, where economic or ideological factors take precedence over nutritional evidence and equity [105]. It is essential to critically assess their role in both human and animal nutrition, while also considering regulatory challenges, cultural acceptability, the presence of allergens, and the limited evidence on their long-term effects. The growing focus on proteins based on insects, microalgae, or fermentation should not replace the development of comprehensive food policies, especially in contexts where food insecurity is a pressing reality. FAO can play a key role by promoting standards based on sound science, strengthening institutional capacities, and preventing the narrative about alternative proteins from devolving into technocratic solutions disconnected from social realities. Finally, a global harmonization of regulations is necessary; however, it must guarantee not only food safety and fair trade but also consumer protection, information transparency, and nutritional sustainability. Without these elements, the transition to new protein sources could become an empty promise rather than a real solution [88,89].

In the face of escalating global challenges—climate change, geopolitical instability, and widening inequalities—renewed efforts to eradicate hunger and malnutrition are not just necessary but urgent. However, such efforts must move beyond generic food security frameworks and engage critically with the nutritional quality of the diets that populations consume. Given the wide variability in AA composition and protein digestibility across food sources, protein quality assessment is not a technical afterthought but a cornerstone of evidence-based food policy [16]. Despite a longstanding acknowledgment of its importance, protein quality remains under-assessed and poorly integrated into many public health strategies. This disconnect undermines the development of effective nutrition education, health promotion, and food-based dietary guidelines (FBDG). Worse still, policies that fail to consider protein bioavailability and digestibility often perpetuate the illusion of adequacy in diets that are nutritionally deficient in practice, particularly in low-resource settings [106,107].

While multilateral institutions, such as the FAO, the International Atomic Energy Agency (IAEA), and other agencies, continue to update methods for protein quality evaluation—including digestibility databases and isotopic techniques—these tools will remain underutilized unless embedded within institutionally supported, context-specific strategies. Relying on global averages or outdated recommendations can lead to inappropriate dietary guidance, especially when applied indiscriminately across culturally and nutritionally diverse populations [16]. Moreover, food-based nutritional guidelines must go beyond promoting balanced diets to explicitly address the limitations of traditional plant-based diets in meeting essential AA. Without clear, science-backed strategies for targeted AA supplementation or food fortification, these guidelines risk reinforcing nutritionally inadequate systems—particularly for vulnerable groups such as children, older, or the food-insecure. Ultimately, the achievement of Sustainable Development Goals and 2030 nutrition targets hinges not just on access to food but on access to high-quality, bioavailable nutrients [16,108].

## 5. Future Directions

In the coming years, PN research should focus on advanced genetic and metabolic phenotyping, incorporating omics- or multi-omics-based approaches. These include high-resolution, high-density analysis of the genome, transcriptome, proteome, metabolome (including epigenetic marks), and microbiome from genetic and functional perspectives. This comprehensive approach enables us to have a more precise characterization of the physiological state of individuals and their response to different dietary patterns. While it is currently technically possible to phenotype individuals using these data, understanding how these markers relate to health or disease progression remains a challenge, especially in pediatric populations, where developmental processes add biological complexity. Although the full integration of these data into neural networks still presents limitations, the use of artificial intelligence tools for their aggregation, analysis, and interpretation represents a crucial advance [108].

In this context, proteomics—which enables large-scale protein analysis in cells, tissues, or biological fluids—not only benefits the discovery of new proteins but will also enable the identification and quantification of bioactive proteins and peptides, as well as the assessment of their nutritional bio-efficacy [60,109]. Focusing attention on the emerging field of omics is not only a technical issue but a strategic one. It will enable us to understand how protein intake influences health, development, and the onset of diseases. This line of research is particularly relevant for children and adolescents, where nutritional requirements are high and dynamic, and where differences in access to, and the quality of, protein sources have clear socioeconomic implications. Integrating omics data into PN could, in the long term, close health gaps and contribute to more effective, equitable, and evidence-based interventions.

Additionally, mass spectrometry (MS)-based food proteomics has established itself as a highly accurate tool for efficiently identifying and quantifying thousands of proteins and peptides. Its application is key in food quality, authenticity, and safety studies, where two complementary approaches exist. First, discovery proteomics enables the analysis of the entire food proteome to identify protein or peptide biomarkers using a bottom-up approach. In this method, proteins are digested into peptides (e.g., with trypsin) and then analyzed by MS to obtain a detailed profile of the compounds present. Targeted proteomics then focuses on the quantitative detection of previously identified biomarkers, whether in food or biological samples, with high sensitivity, precision, and reproducibility [110]. This approach is essential for confirming findings and applying technology in regulatory, clinical, or industrial contexts [60].

Interestingly, the bioinformatic integration of proteomics with other omics platforms, such as foodomics, expands its potential by enabling the prediction of nutritional functionality and protein allergenicity in novel foods. This discovery is particularly relevant in the evaluation of genetically modified crops, where exhaustive characterization is required to ensure their food safety and social acceptance. Within nutriproteomics, proteomics techniques enable the examination of dietary effects on protein expression at the molecular level, including interactions with other nutrients regarding bioavailability, biological activity, and stability. This method simplifies the detection and measurement of bioactive peptides and food-derived proteins. These substances can regulate key processes, such as growth and metabolic balance, and, in some cases, trigger allergic reactions [60]. Together, these advances highlight the importance of incorporating proteomic methodologies in the design of safer, more functional, and personalized food systems. However, widespread adoption still requires greater technical accessibility, institutional capacity, and global regulatory harmonization efforts.

In the field of PN, one of the most relevant cellular mechanisms is the mammalian target of rapamycin complex 1 (mTORC1), recognized as a key AA sensor and master regulator of body growth and neurocognitive development. mTORC1 is part of a system composed of two multiprotein complexes, comprised of different proteins that interact specifically to control cell growth, motility, and metabolism, each sensitive to unlike stimuli. mTORC1 is triggered by nutrient levels, especially amino acids, whereas mTORC2 responds to growth factors via the phosphoinositide 3-kinase pathway [39,111]. When AA levels are adequate, mTORC1 adopts an active conformation that stimulates anabolic pathways, such as protein, lipid, and nucleotide synthesis, while inhibiting catabolic processes, such as autophagy, which promotes linear growth. In contrast, AA deficiency prevents mTORC1 activation, suppressing the biosynthesis of essential macronutrients, activating autophagy in an unregulated manner, and halting cell growth [39].

It should be emphasized that this suppression cannot be counteracted by other signals, such as hormonal factors or cell energy status, which highlights the centrality of AA as metabolic regulators. In addition, another critical pathway comes into play in situations of protein deficiency; the GCN2 (general checkpoint non-derepressible 2) system acts as a cellular sensor of AA deficiency. Its activation blocks mRNA translation, induces autophagy, and inhibits mTORC1, thus perpetuating a catabolic state that compromises cell growth and renewal [39]. From an endocrine perspective, dietary proteins may stimulate elevated levels of insulin, IGF-1, and free triiodothyronine, hormones that promote bone and tissue growth. mTORC1, specifically, is critical for bone growth, where it stimulates endochondral ossification. Nutrients such as leucine and glutamine—both abundant in eggs—appear to play a prominent role in activating the mTORC1 pathway in muscle cells, promoting protein synthesis in skeletal muscle [80]. This body of evidence underscores the importance of adequate, high-quality protein intake during critical developmental stages and reveals the complex molecular interactions that mediate nutritional effects on growth.

## 6. Conclusions

Animal proteins typically have a higher biological value due to their complete amino acid profiles. However, plant-based proteins can also support healthy growth if they are carefully selected and combined. Although the transition to plant-based diets is encouraged to reduce the environmental impact of livestock farming worldwide, it is essential to ensure that individuals receive an adequate quantity of protein depending on their specific characteristics, including age, sex, health status, illnesses, and sociocultural factors. It is indispensable for pediatric populations to obtain all nine essential amino acids and to receive appropriate vitamin and mineral supplementation, depending on whether they follow an animal-based, plant-based, or mixed diet.

Public health strategies must be context-specific, prioritizing nutritional adequacy and equity over generalized dietary ideologies. Ignoring socioeconomic and nutritional realities risks implementing misguided policies that may cause more harm than good. Dietary guidelines and food system interventions should address not only the long-term risks of excess in affluent settings but also the immediate risks of deficiency in vulnerable populations. International recommendations on protein intake must be critical, scientifically sound, and socially responsive. Neglecting these dimensions risks reinforcing nutritional inequalities and undermining the impact of food and health interventions worldwide. Global institutions must critically reassess whether their current efforts are sufficiently equipped to meet this standard—or risk repeating cycles of well-intentioned but ineffective interventions. Significant challenges remain in translating this knowledge into personalized nutrition recommendations, particularly in pediatric populations and in settings of nutritional vulnerability.

## Figures and Tables

**Table 1:** Comparison of protein sources regarding their biological value, digestibility, net protein utilization, and amino acids content.

Protein Source	Biological Value	Digestibility (%)	Net Protein Utilization	Amino Acid Content	Tips	Reference
Egg (whole)	100	~98%	94	Complete; rich in leucine, lysine, methionine	Gold standard for protein quality	[22,23]
Whey protein	104–110	~99%	92	High in BCAAs (leucine esp.); fast digesting	Rapid absorption, ideal for muscle synthesis	[24,25]
Casein (milk)	~77	~97%	76	Complete; slow digestion profile	Good for sustained amino acid release	[24,25]
Milk (whole)	91–93	100	82	Complete, rich in lysine	High total protein content	[20,24]
Beef	80	~94%	78	Complete; high in histidine and lysine	Also contains creatine and heme iron	[24,26]
Chicken	~79	~95%		Complete; rich in lysine and leucine	Lean meat, low fat	[27]
Fish (e.g., salmon)	~83	~94%		High in lysine, methionine, taurine	Also provides omega-3 fatty acids	[3,27]
Soy protein	~74	~91%	61	Complete, but lower in methionine	Best plant-based complete protein	[24,28]
Pea protein	~65	~89%		High in lysine, low in methionine	Often used in vegan supplements	[22]
Rice protein	~59	~88%		High in methionine, low in lysine	Often combined with pea for a complete profile	[29]
Wheat protein (gluten)	~54	~86%	67	Low in lysine and threonine	Incomplete on its own	[24,28]
Lentils	~49	~85%	27–33	High in lysine, low in sulfur-containing AAs	High in fiber, best when combined with grains	[30]
Spirulina	~50–60	~84%		Rich in glutamic acid, contains all essential AAs	Algae source, nutrient-dense	[31]

Legend: BCAA: branched-chain amino acids (leucine, isoleucine, and valine).
